# Biases in ecological research: attitudes of scientists and ways of control

**DOI:** 10.1038/s41598-020-80677-4

**Published:** 2021-01-08

**Authors:** Elena L. Zvereva, Mikhail V. Kozlov

**Affiliations:** Department of Biology, University of Turku, 20014 Turku, Finland

**Keywords:** Ecology, Psychology

## Abstract

The properties of the human mind affect the quality of scientific knowledge through the insertion of unconscious biases during the research process. These biases frequently cause overestimation of the effects under study, thereby violating the reproducibility of the research and potentially leading to incorrect conclusions in subsequent research syntheses. We explored the level of knowledge about biases and attitudes to this problem by analysing 308 responses of ecology scientists to a specifically developed survey. We show that knowledge about biases and attitude towards biases depend on the scientist’s career stage, gender and affiliation country. Early career scientists are more concerned about biases, know more about measures to avoid biases, and twice more frequently have learned about biases from their university courses when compared with senior scientists. The respondents believe that their own studies are less prone to biases than are studies by other scientists, which hampers the control of biases in one’s own research. We conclude that education about biases is necessary, but not yet sufficient, to avoid biases because the unconscious origin of biases necessitates external intervention to combat them. Obligatory reporting of measures taken against biases in all relevant manuscripts will likely enhance the reproducibility of scientific results.

## Introduction

The properties of the human mind can affect the quality of the research through insertion of a number of biases, which are generally defined as systematic errors in results or inferences that favour one outcome over others^1^. All phases of scientific study are prone to biases^2^. One of the widely known biases is publication bias, which has a recognized influence on the scientific knowledge^3–5^. By contrast, the occurrence and importance of biases introduced at pre-publication stages of research have received considerably less attention (but see^6–8^). This is especially true for confirmation or observer bias, which is defined as a tendency to search for, interpret and favour information in a way that confirms one’s pre-existing hypotheses or beliefs^9,10^.

Confirmation bias occurring due to unconscious psychological processes^11^ is usually demonstrated by comparing the results of studies conducted blindly and not blindly^12,13^. Blinding, known as an important measure of minimizing unconscious biases, is a routine procedure in medical studies^14,15^. At the same time, blinding is only rarely reported in life science studies, e.g. in ecological and evolutionary papers^16^, and the outcomes of blind and non-blind methods have rarely been compared within this research domain^17^. When this comparison has been performed, the lack of blinding with respect to the hypothesis being tested or the treatment condition of a sample usually resulted in overestimation of the effects in both primary studies and meta-analyses^8,13,18^. Similarly, a lack of true randomization in the selection of experimental units (i.e. the smallest entities that could be subjected to an intervention independently of each other) causes a considerable overestimation of the effects under study^8^, indicating that cognitive biases influence the outcomes of the studies already in the planning stage. The increasing use of quantitative research synthesis in ecological and environmental sciences^19,20^ makes the consequences of biases in empirical studies especially severe, because the effects of these biases are accumulated and generalized when combined in meta-analyses.

Thus, the existing life science knowledge is likely to be considerably biased, and measures to combat cognitive biases affecting research should be urgently developed and implemented as a part of activities aimed at the general improvement of transparency and reproducibility in science^21–23^. The elaboration of such measures requires information regarding the current level of awareness about biases among scientists and about scientists’ understanding of the danger of biases in their research domain in general and in their own research in particular. As neatly stated by one of our anonymous respondents, “the most dangerous bias is if we believe there is no bias”*.*

In the present study, we evaluate the extent of the knowledge held by ecology scientists regarding different biases and we explore the attitudes of scientists to this problem by analysing the information obtained from a specifically developed questionnaire. In particular, we wanted to answer the following questions: (i) To what extent are ecology scientists aware of the occurrence and importance of biases during the research process? (ii) How highly do ecology scientists evaluate the impact of biases on different stages of the research process, on different fields of life sciences and methods of data collection, on science in general, on their particular research field, and, finally, on their own studies? (iii) How much do ecology scientists know about the different ways to avoid bias? (iv) Do scientists’ knowledge about biases and their attitude to biases depend on the stages of their scientific careers, their genders or their affiliation countries?

For our study we selected the domain of Ecology and Environment (Ecology hereafter), which was earlier found to be prone to a great number of biases^7–9,12,13,16,18^ due to the wide variety of methods used, including (but not limited to) observations, field and laboratory experiments, and modelling. However, many results obtained for Ecology are likely to be valid for other domains of science because many types of biases in ecological research are explained by unconscious processes in the human mind^10,11^ that do not depend on a particular area of research.

## Results

A total of 779 persons opened the survey, 486 persons started responding to it, and 308 persons from 40 countries submitted their responses. Seven of these 308 respondents had never heard about biases, and the remaining 301 persons (i.e. 38.6% of those who opened the survey) answered our questions about their attitude to biases.

Nearly all (98%) scientists who responded to our survey were aware of the importance of biases in science. Among these, 33% reported ‘very well’ for their awareness on this topic, 52% classified their awareness as ‘well’ and 13% as ‘poor’. Most of respondents learned about biases from their university courses (36%), from contacts with colleagues (22%) or from scientific literature (20%). Among the different kinds of biases, the best known was observer/observation bias (82%), followed by publication bias (71%) and selection bias (70%); confirmation, reporting/presentation, researcher, measurement, geographic and funding biases were known to 50–60% of respondents (see Appendix S1). Among the seven suggested definitions of biases (see Appendix S1), ‘the tendency to search for, interpret, and publish information in a way that confirms one’s pre-existing beliefs or hypotheses’ corresponded to the understanding of biases by 80% of the respondents, and ‘preferential publication of statistically significant results’ was ranked the second, being selected by 61% of respondents.

In the opinion of our respondents, the stages of scientific research differed in their susceptibility to biases (χ^2^_20_ = 90.0, P < 0.0001) and were ranked as follows, from greatest to least bias susceptibility: interpreting the results > planning/designing the study > publishing the outcomes > reporting the outcomes > analysing the results > implementing the study. Similarly, the types of publications were considered prone to biases to different extents (χ^2^_16_ = 86.8, P < 0.0001) and were ranked as follows: narrative reviews > studies based on observational data > studies based on modelling > studies based on experiments > meta-analyses.

**Figure 1:**
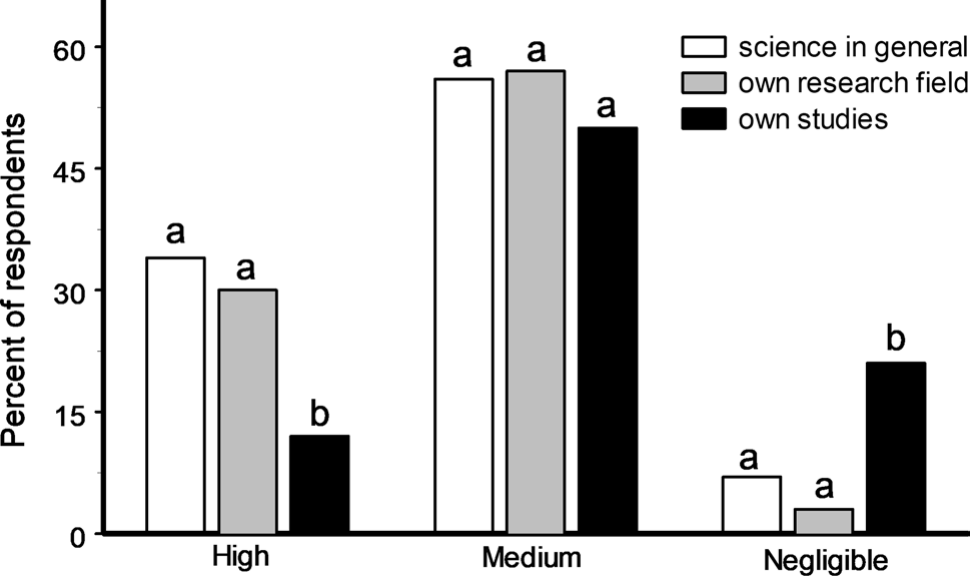
Impact of biases on science in general, on one’s own research field and on one’s own studies, as estimated by 301 respondents. Bars marked with different letters differ from each other at P < 0.05 within each group (χ^2^ test).

Most scientists thought that the severity of the impact of biases on science in general and on their particular research field was medium or high, and a few considered that it was negligible. At the same time, our respondents estimated the impact of biases on their own studies as high almost three times less frequently and as negligible seven times more frequently when compared with their estimates of the impact of biases on other studies within their own research field (Fig. 1).

The respondents considered the most important ways to avoid biases (see Appendix S1) to be reporting all results, not only statistically significant ones (89% of respondents), checking for repeatability of all measurements (78%), performing a random choice of experimental units (78%) and using blinding (70%). At the same time, 15% of respondents believed that a haphazard (i.e., neither systematic nor random in a strict sense, and therefore subjective and prone to biases) choice of experimental units could also help to avoid biases. Most researchers reported that they were thinking about biases that could affect the outcomes of their own studies (81%) and that they planned and implemented particular measures to avoid biases in their research (75%), but only 61% of the respondents reported these measures in their publications.

As expected, the scientific productivity and teaching activity depended on the stage of the career. Senior scientists published more scientific papers during the three years preceding their responses to our questionnaire than did the mid-career and early career scientists (12.2, 8.6 and 3.8 papers, respectively). Senior and mid-career scientists reviewed more manuscripts during the same period (12.3, 12.7 and 4.7 manuscripts, respectively) and were more frequently involved in teaching than were the early career scientists (80, 82 and 55%, respectively).

**Figure 2:**
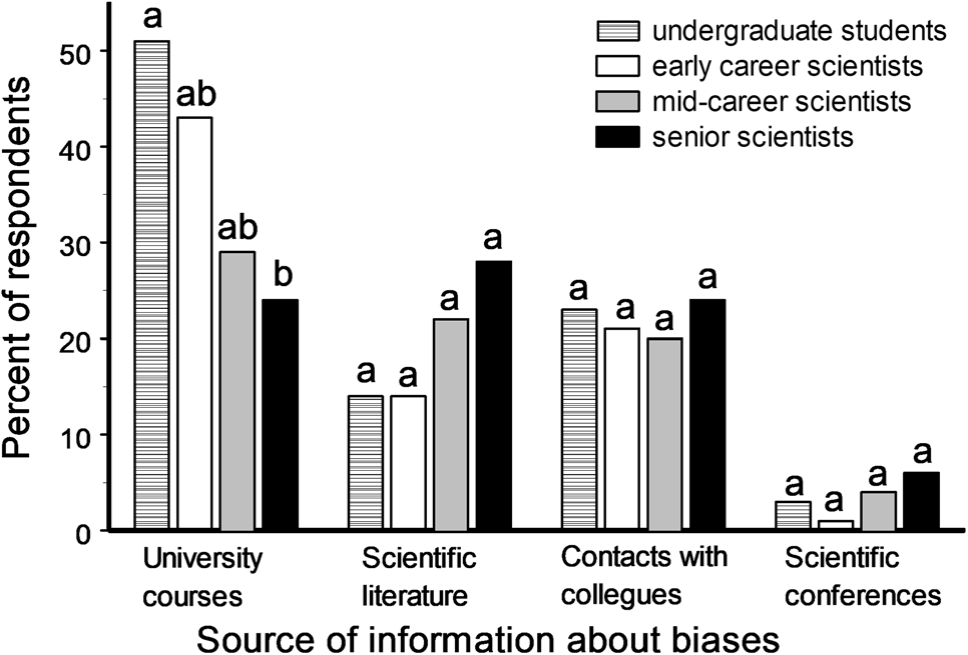
Sources of the first information about biases in relation to the stage of respondent’s research career, as reported by 35 undergraduate students, 122 early career scientists, 49 mid-career scientists and 95 senior scientists. Bars marked with different letters differ from each other at P < 0.05 within each group (χ^2^ test).

**Figure 3:**
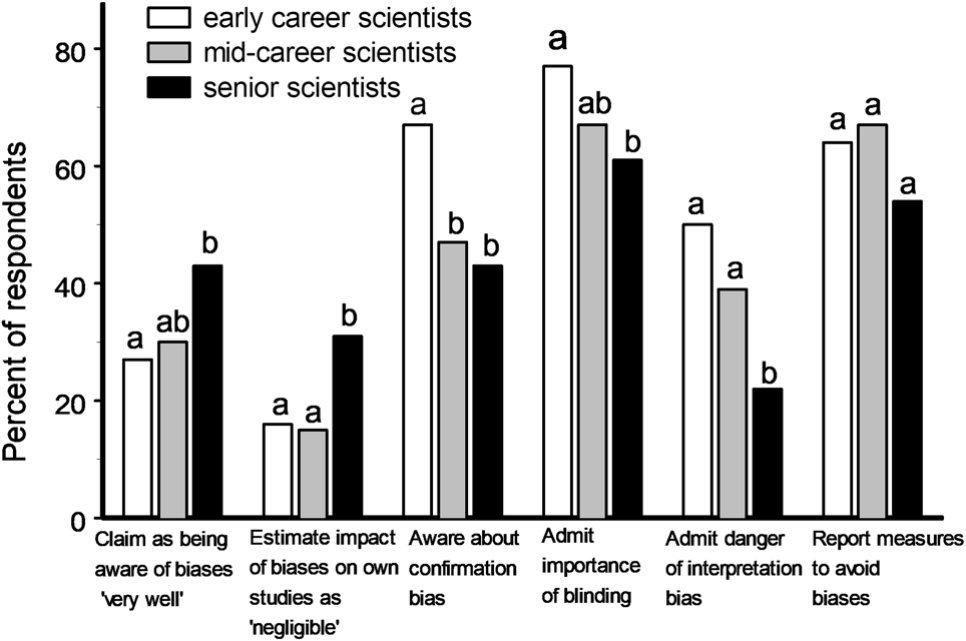
Selected characteristics of the respondents’ attitudes to biases in relation to the stages of their research careers, as reported by 124 early career, 50 mid-career and 97 senior scientists. Bars marked with different letters differ from each other at P < 0.05 within each group (χ^2^ test).

The understanding of biases did not change with the career stage, as indicated by the similar selection of different definitions of biases by early, mid-career and senior scientists (χ^2^_12_ = 6.71, P = 0.88). A higher proportion of undergraduate students and early career scientists had learned about biases from university courses when compared with mid-career and senior scientists, whereas the more advanced career group had learned about biases mostly from the scientific literature (Fig. 2). Early career scientists were aware of a larger number of bias types (Appendix S1) when compared with either the mid-career (S = 57, P = 0.005) or the senior scientists (S = 72.5, P = 0.0001). In particular, a larger fraction of early career scientists, compared with senior scientists, were aware of such important biases as confirmation bias (Fig. 3), observer bias (85 and 75%, respectively; χ^2^_1_ = 4.34, P = 0.04), selection bias (77 and 64%, respectively; χ^2^_1_ = 4.33, P = 0.04) and cognitive bias (34 and 21%, respectively; χ^2^_1_ = 4.74, P = 0.03).

Early career scientists generally gave for the impact of biases on all stages of the research process a higher rating than the senior scientists did (S = 10.5, P = 0.03), with greatest differences seen in the percentage of respondents who admitted high impacts of interpretation bias (Fig. 3) and of reporting bias (34 and 22%, respectively; χ^2^_1_ = 4.30, P = 0.04) in ecological research. Among methods that would allow the avoidance of biases, early career scientists mentioned blinding more frequently than senior scientists did (Fig. 3), while the importance of other methods was similarly appreciated by both groups of researchers. At the same time, senior scientists declared more frequently than early career scientists that they were ‘very well’ aware of biases, and the senior scientists estimated the impact of biases on their own studies as ‘negligible’ twice as frequently than the early and mid-career scientists did (Fig. 3).

The proportion of women among our respondents declined with the duration of their professional activity, from 57% of early career scientists to 38% of mid-career scientists, and finally dropped to 27% of senior scientists (χ^2^_2_ = 19.6, P < 0.0001). This proportion was greater in high GDP countries than in low GDP countries (52 and 28%, respectively; χ^2^_1_ = 14.8, P = 0.0001). Female respondents had published fewer papers than male respondents during the past three years (6.0 and 9.1, respectively; χ^2^_1_ = 11.1, P = 0.026) and females were less involved in teaching than male respondents across all stages of their careers (61 and 78%, respectively; χ^2^_1_ = 11.9, P = 0.0006).

Male and female respondents significantly differed in their attitudes towards biases. More men than women claimed that they were ‘very well’ aware of biases (39% and 27%, respectively; χ^2^_1_ = 5.43, P = 0.02). At the same time, female scientists gave higher ratings for the impacts of biases on the different stages of research (S = 10.5, P = 0.03) and on the different fields of science (S = 18, P = 0.008) than male scientists did.

**Figure 4:**
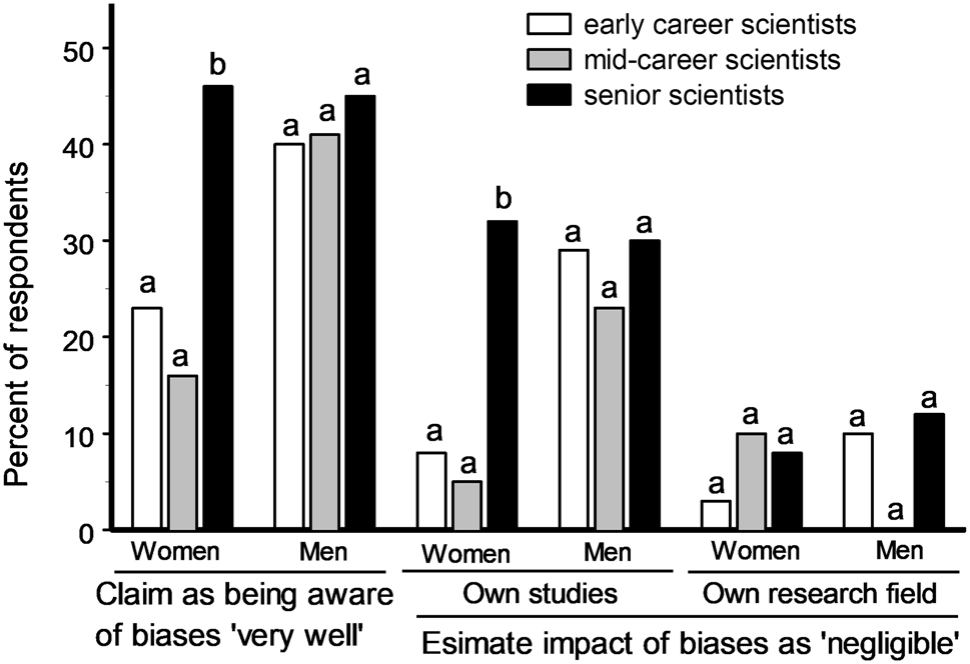
Self-estimates of the awareness about biases and of their impact on one’s own studies and on other studies in one’s own research field in female and male scientists in relation to the stages of their research careers, as reported by (women/men) 70/50 early career, 19/27 mid-career and 26/65 senior scientists. Bars marked with different letters differ from each other at P < 0.05 within each group (χ^2^ test).

Female and male respondents had similar assessments of the severity of the impact of biases on science in general and on their particular research field (χ^2^_1_ = 7.54, P = 0.06 and χ^2^_1_ = 5.85, P = 0.12, respectively), but female respondents gave much more critical evaluations of the impact of biases on their own studies: 16% of women and 8% of men assessed this impact as ‘high’ (χ^2^_1_ = 3.98, P = 0.046), whereas 13% of women and 27% of men considered it ‘negligible’ (χ^2^_1_ = 8.59, P = 0.003) (Fig. 4). The latter result mostly reflects gender differences within the group of early career and mid-career scientists, while the attitude of senior scientists to biases in their own studies did not differ between women and men (Fig. 4).

**Figure 5:**
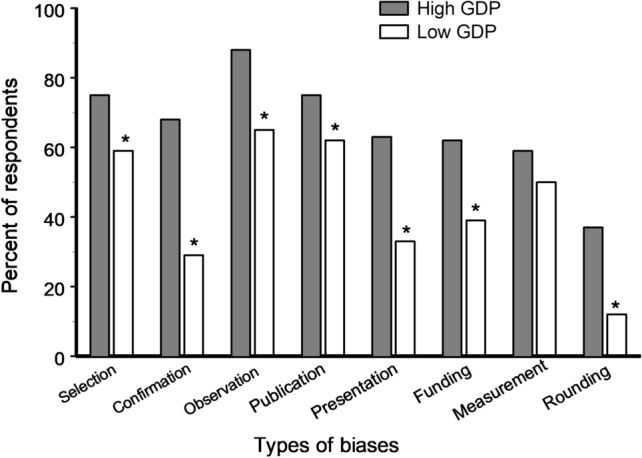
Percent of respondents from high and low GDP countries (219 and 82 scientists, respectively) who were aware of the most commonly known biases. Asterisks indicate significant (P < 0.05) differences between high and low GDP countries (χ^2^ test).

Respondents from high GDP countries were aware of a greater number of biases than were respondents from low GDP countries (8.5 and 5.9 biases per person, respectively; S = 75.5, P < 0.0001; Fig. 5). Within the high GDP countries, respondents from the USA declared themselves ‘very well’ aware of the importance of biases more frequently than European scientists did (42 and 24%, respectively; χ^2^_1_ = 8.30, P = 0.004), and the American respondents knew about a greater number of bias types when compared with the respondents from Europe (9.1 and 7.5 biases per person, respectively; S = 63.0, P = 0.001).

Respondents from high GDP and low GDP countries estimated the impact of biases as high equally frequently with respect to science in general (32 and 40%, respectively; χ^2^_1_ = 1.57, P = 0.21), their particular research field (27 and 38%, respectively; χ^2^_1_ = 3.67, P = 0.06) and their own studies (11 and 17%, respectively; χ^2^_1_ = 2.39, P = 0.12). Similar proportions of respondents from both groups evaluated the impact of biases on their own studies as negligible (20 and 21%, respectively; χ^2^_1_ = 0.02, P = 0.90).

**Figure 6:**
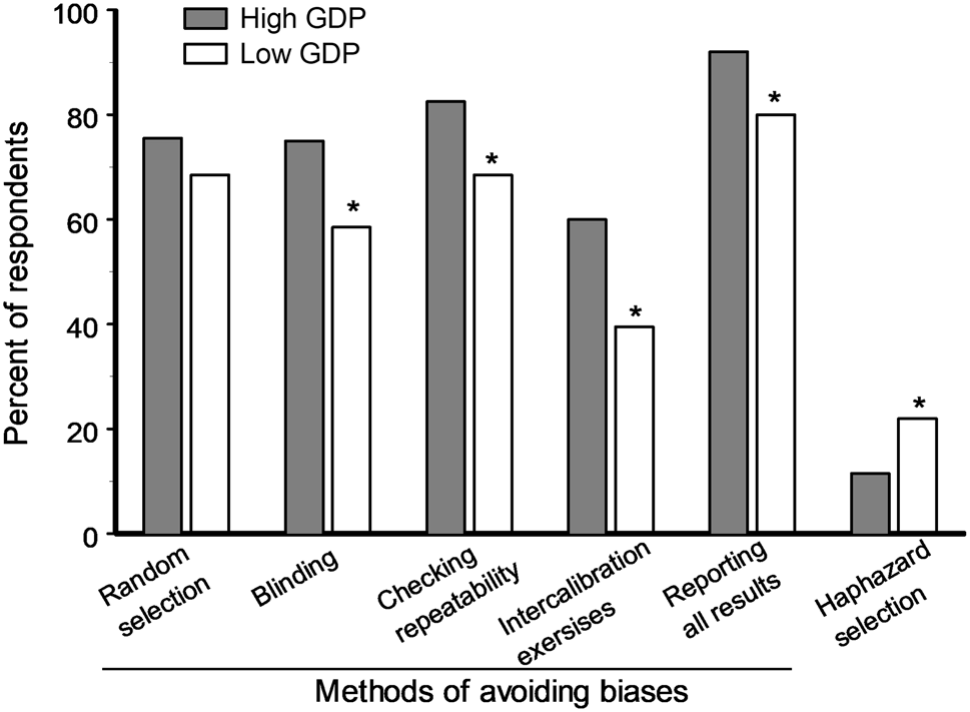
Percent of respondents from high and low GDP countries (219 and 82 scientists, respectively) who knew different methods for avoiding biases and who erroneously thought that haphazard selection also helps to avoid biases. Asterisks indicate significant (P < 0.05) differences between high and low GDP countries (χ^2^ test).

Respondents from high GDP countries were more aware of four (of the five suggested) methods for avoiding biases when compared with the respondents from low GDP countries (Fig. 6). At the same time, haphazard selection of experimental units was considered as a measure to avoid biases twice more frequently by respondents from low GDP countries than by respondents from high GDP countries (Fig. 6).

Similar proportions of scientists from high and low GDP countries (37 and 32%, respectively; χ^2^_1_ = 0.73, P = 0.39) learned about biases from their university courses, but a significantly lower proportion (15%) of scientists from Eastern Europe and Russia obtained information about biases in this way when compared with this proportion (40%) of scientists from the rest of the world (χ^2^_1_ = 4.94, P = 0.02).

## Discussion

### Who responded to our survey?

The proportions of people who opened the survey but did not complete it and who submitted the survey but had never heard about biases jointly yielded a conservative estimate (62%) of the proportion of scientists who are not aware of and/or are not concerned about the problem of biases, and would therefore be unlikely to account for biases in their own studies. This value indicates that awareness and/or understanding of the importance of biases and, consequently, knowledge about measures for their avoidance are generally low among ecology scientists.

Our findings correspond to the estimates published earlier: in the domain of ecology, evolution and behaviour, only 13% of studies potentially influenced by observer bias are conducted in a blinded way^16^, and in the particular area of herbivory studies, this proportion is as low as 10%^8^. Therefore, when discussing our results, we keep in mind that they are prone to non-response bias, and that scientists who are aware of and who care about biases are overrepresented in our sample. As a result, our data evidently overestimate the average level of awareness of bias-related issues among ecology scientists. This was intuitively understood by many of our respondents who suspected that the results of our survey would be biased (Appendix S1). However, this bias may only influence our conclusions on the level of awareness and concern about biases in scientific community.

### “Why do you see the speck that is in your brother's eye, but do not notice the log that is in your own eye?”: Bias blind spot in scientific research

Our respondents estimate the risk of biases in their own studies as much lower than in science in general and in studies by other scientists working in the same research field (Fig. 1). This asymmetry is known in psychology as a bias blind spot—the tendency of people to believe that they are less susceptible than other people to nonconscious predispositions and cognitive biases^24–26^.

We discovered a strong bias blind spot in scientific research and found that the strength of this bias varies with gender and with the stage of professional career: the difference in the estimate of biases in one’s own vs anotherʼs studies is two times larger in men than in women and two times larger in senior scientists than in early career scientists. At the same time, although the overall knowledge about biases differs considerably between high and low GDP countries (Figs. 5, 6), the strength of the bias blind spot is similar in these countries, indicating that an ability to critically evaluate one’s own research reflects basic characteristics of the human mind and does not depend on a person’s cultural or economic background.

The gender differences in the bias blind spot found in our study are consistent with the outcomes of earlier gender studies, which found that men usually overestimate their intelligence and abilities relative to their objective measures to a greater extent than women do^27–29^. In addition, more female than male scientists classify different research fields and stages of the research as being prone to biases, but women are more modest than men in evaluating their own knowledge about biases. Thus, female scientists admit to shortages in their knowledge more often, and they recognize the danger of biases better than male scientists do. When combined with the gender difference in the bias blind spot, this result indicates that female scientists in general are less susceptible to biases than are male scientists, at least within the field of ecology. Involving more women in teaching and research could therefore improve the overall attitude of scientists to the problem of biases.

Intriguingly, the asymmetry in estimating impact of biases on one’s own and another’s research (the bias blind spot) considerably increases in women senior scientists compared with women in earlier career stages and becomes as high as that in men (Fig. 4). This trend may be explained by a growth in self-esteem with age and experience^30^, but another possibility is that only women with high self-confidence and self-esteem can reach high career levels in science. The latter suggestion is indirectly supported by the steady decrease in the proportion of women within our respondent group with increasing career stage. Thus, senior female scientists become as susceptible to biases in their research as male scientists.

In the experiments examining whether or not individuals see the existence of cognitive biases much more in others than in themselves^24^, the respondents (psychology students) insisted that their self-assessments were accurate and objective even if they knew how their responses could have been affected by a relevant bias. Similarly, many of our respondents claimed that the effect of biases on their own studies was negligible, even when they were well aware of the potential effects of biases on the research process. A striking example showing the impossibility of consciously avoiding some biases in one’s own research was provided by Kozlov et al.^18^: in that study, the values of site-specific plant damage by insects estimated in a purposely unbiased way (but not blindly) were much higher than the values obtained in a truly blinded way. However, early career scientists, who have better education concerning biases, are less prone to the bias blind spot when compared with scientists at higher stages in their scientific career. Thus, knowledge about biases may, potentially, reduce the risk of biases in one’s own research. Nevertheless, due to the unconscious nature of cognitive biases, merely possessing knowledge about biases is not sufficient to avoid them.

We detected ‘bias blind spot’ and explored the sources of variation in its strength among ecology scientists. However, we suggest that this phenomenon is widespread in many research domains, because it is based on unconscious psychological processes characteristic for every human mind.

### Sources of variation in knowledge of and attitude to biases among scientists

Scientists from low GDP countries generally show weaker knowledge about biases and about the methods to avoid them than do scientists from high GDP countries. Importantly, more than 20% of the scientists from low GDP countries think that haphazard selection of experimental units would help to avoid bias, which means that many scientists do not distinguish between random and haphazard selection. The frequent misuse of term ‘randomʼ has been demonstrated earlier by Zvereva and Kozlov^8^ in a particular area of ecological studies. This mistake is very dangerous, because haphazard selection of experimental units is very prone to cognitive biases and leads to considerable overestimation of the effect when compared with results obtained with selection based on randomization procedures^8^. These findings hint at a generally lower level of education with regard to research methodology in low GDP countries compared with high GDP countries, and especially in Eastern Europe and Russia, where only 15% of our respondents learned about biases from their university courses.

We found that early career scientists are more aware of various biases and about measures to avoid them, in particular blinding, when compared with mid-career and senior scientists (Fig. 3). This difference is of critical importance because blinding is vital for avoiding a severe impact of cognitive biases in many areas of biological sciences, and in ecology in particular^8,16,17,31^. This pattern may reflect quite recent improvement in education with respect to biases, because mid-career scientists (10–20 years beyond graduation) had learned about biases from their university courses less frequently when compared with the younger scientists.

The greater knowledge about biases among early career researchers is accompanied by better acknowledgment of the risk of different types of biases on different stages of research than is observed for senior scientists, whereas self-declared knowledge follows the opposite pattern and increases with the stage of career (Fig. 3). This gap between objective and self-declared knowledge in senior scientists may reflect changes in general self-esteem with age and with success in professional careers^30^. At the same time, more senior scientists believe that they are not affected by cognitive biases (Fig. 4). These findings jointly indicate that senior scientists, who are the most influential in the scientific community in terms of publications, reviewing of manuscripts and teaching, are the most prone to unconscious biases. We do not know, however, whether early career scientists, who at the moment appear the least susceptible to biases, will maintain their current concerns regarding biases as their scientific careers advance, or whether the differences between age groups will persist in future due to growth in self-esteem with age and to selection for scientists with high self-confidence and self-esteem.

### How to minimize impacts of cognitive biases on science?

Low knowledge of biases leads to underestimation of the risks associated with these biases and, in turn, to ignoring the measures needed to avoid them in a scientistʼs own research. Although we observed some improvement in the knowledge about biases in the younger generation of scientists, the situation is still far from being perfect, especially when taking into account the great proportion of scientists who do not know anything about biases in research or who are not concerned about bias impacts on the outcomes of scientific research. Therefore we suggest that a course on research methodology should be obligatory in the academic education of biology students and that this course should include sufficient information about cognitive biases in research.

Most of the biases that influence research occur due to the inherent thinking errors that humans make when processing information, and these errors prevent an accurate understanding of reality. Cognitive biases are unconscious, which means that simply being aware of the existence and importance of biases is not sufficient to avoid them and that an external intervention is necessary to combat biases during the research process. We therefore suggest including in the author’s checklists of all journals in the field of ecology, evolution and environmental sciences a requirement that all measures taken against biases be described in each submitted manuscript. The need to check for these requirements should also be included in the reviewer’s checklist. This idea is not completely new, because some high impact journals (for example, Nature journals) have already included questions about measures of avoiding biases (e.g. randomization and blinding) into the author’s checklist. To enhance the quality of life science research in general, other journals are recommended to join in this initiative. This request would stimulate scientists to learn more about potential biases related to their research and about methods for bias avoidance. We also recommend that the authors of future meta-analyses consider the presence/absence of randomisation and blinding, whenever appropriate, as important explanatory variables. Our study clearly shows that if we want to improve the methodology of ecological research, incorporating these measures is unavoidable.

Biases occurring at different stages of research may sometimes appear to be the reason for the lack of reproducibility—an issue which has recently received increasing attention from the scientific community^32,33^. Measures of improving transparency and openness in ecology and evolution^22,23^ may appear insufficient to ensure reproducibility of the research^33^. In particular, this is because the impacts of biases on the primary data collected non-blindly and without proper randomization procedures would depend greatly on the personality and individual preferences of the researcher. These data would therefore be much less likely reproducible compared to blindly collected data.Therefore, obligatory measures to avoid cognitive biases would likely enhance the reproducibility of scientific results.

## Conclusions

1. The awareness and/or understanding of the importance of biases—and, consequently, knowledge about measures for their avoidance—are generally low among ecology scientists.
2. Low knowledge of biases leads to underestimation of the risks associated with these biases. This, in turn, leads to a tendency to ignore the measures needed to avoid these risks in a scientistʼs own research.
3. Early career scientists are more concerned about biases and know more about measures to avoid biases compared with senior scientists. Thus, the most influential group of scientists is the one most prone to biases.
4. Scientists believe that their own studies are less prone to biases than are studies by other scientists (“bias blind spot”), and this hampers the control of biases in one’s own research.
5. Obligatory reporting of the measures taken against biases in all relevant manuscripts will likely enhance the reproducibility of scientific results.

## Methods

The study was conducted as a questionnaire-based survey implemented using the Webropol 3.0 tool (www.webropol.com). Both the questions and the suggested answers (e.g. definitions of biases, types of biases, measures to avoid biases) were developed or identified based on textbook and scientific literature review, as well as using a free web search for the keyword ‘bias’.

In total, 12 questions were aimed at estimating the level of knowledge about biases among the ecology scientists, the level of concern they have about biases and how highly they evaluate the impact of biases on ecological science (Appendix S1). Five of these 12 questions required a single choice among several options, whereas four questions (about known types of biases or measures to avoid them) allowed multiple selections. Three more questions aimed at revealing the opinions of the respondents about the severity of the impact of biases on different areas of science, on stages of research and on types of scientific publications used 5-point Likert-type scales.

We estimated the self-declared awareness of the respondents about the importance of biases by requesting them to attribute their knowledge to one of four levels (Appendix S1). The conclusions regarding the objective knowledge were derived from the numbers of different biases and the methods for avoiding biases which the respondents marked as known to them. The survey also contained 9 questions characterizing our respondents (gender, age, affiliation, career stage, scientific productivity and involvement in teaching). The last question asked the opinion of our respondents regarding the potential of our survey to obtain unbiased information about the study problem. The respondents were also invited to leave their free-style comments on the topic of the survey. In the present study, we used responses to 22 questions (listed in the Appendix S1) of 26 questions that have been included in our survey.

We distributed the first draft of the questionnaire among five of our colleagues, and we accounted for their feedback while preparing the final version. On 21 April 2019, we sent the link to our survey to our collaborators (ca. 40 ecology scientists worldwide), who distributed it further via several mailing lists. On 8 May 2019, when the rate of increase in the number of respondents had declined to 0–1 persons/day, we additionally sent the link to 227 scientists from 13 countries. The names of these scientists were drawn (on a first-found basis) from the ISI Web of Sciences database using a search for papers with the word “ecolog*” in the title and a publication date falling within the year preceding the search date (8 May 2019). The survey was closed on 14 June 2019.

The study was carried out in accordance with “The ethical principles of research with human participants and ethical review in the human sciences in Finland (the Finnish National Board on Research Integrity TENK guidelines 2019)”. According to this document, our research did not require ethical review in Finland. The participation in our study (i.e., responding to an online questionnaire) was voluntary, and the participants were informed that their anonymous answers would be used in scientific research.

For the purposes of our study, we defined the stages of a scientific career as follows: early career scientists (less than 10 years from college/university graduation; 124 respondents), mid-career scientists (10–20 years from graduation; 50 respondents) and senior scientists (more than 20 years from graduation; 97 respondents). We used the responses of undergraduate students (n = 35) in the analysis of sources of information about biases, but we excluded this group from other analyses because their knowledge on and concerns about biases may still change when their education is complete. The question about the respondent's gender included the option ‘prefer not to say’; consequently, 15 respondents who selected this option were excluded from the analysis of gender differences. We used GDP for 2018^34^ as a measure of the standard of living and accepted GDP $30,000 as the borderline between high and low GDP countries (n = 222 and 86 respondents, respectively). We also contrasted Eastern European countries (Poland, Slovakia, Slovenia, Czech Republic) and Russia versus other countries, and USA versus high GDP West European countries.

We used frequency analysis, which employed the chi-square as the test statistics, to compare non-paired groups of data and the Wilcoxon signed rank test to compare paired groups of data. The analyses were performed using either the Webropol statistical tool or SAS version 9.4^35^. All statistical tests were two-sided.

## Supplementary Information

Supplementary Information.

## Data Availability

All data from this study are included in this publication and its Supplementary Material.
