# Supplementary material for: Biases in ecological research: attitudes of scientists and ways of control
Source: Sci Rep. 2021 Jan 8;11:226. doi: 10.1038/s41598-020-80677-4 (PMC7794457; doi:10.1038/s41598-020-80677-4)
Supplement: Supplementary file 1 — Supplementary Information. [file 41598_2020_80677_MOESM1_ESM.docx]

**Biases in ecological research: attitudes of scientists and ways of control**

**By Elena L. Zvereva and Mikhail V. Kozlov**

**Appendix S1: Outcomes of the survey**

**How aware are you of the importance of biases in scientific research?**

| **Awareness level** | **n** | **Percent** |
| --- | --- | --- |
| Very well | 101 | 32.79% |
| Well | 161 | 52.27% |
| Poorly | 39 | 12.66% |
| Not at all | 7 | 2.28% |

**Where did you first learn about biases in scientific research?**

| **The first source of knowledge about biases** | **n** | **Percent** |
| --- | --- | --- |
| Basic university courses/textbooks | 107 | 35.55% |
| Scientific literature | 59 | 19.6% |
| Scientific conferences | 10 | 3.32% |
| Contacts with colleagues | 67 | 22.26% |
| Reviewer’s comments to my manuscript | 8 | 2.66% |
| Other | 11 | 3.65% |
| I do not remember | 39 | 12.96% |

**About which biases in the following list you have ever heard or read in association with scientific research? (Mark all that apply)**

| **Types of biases** | **n** | **Percent** |
| --- | --- | --- |
| Selection bias | 212 | 70.43% |
| Confirmation bias | 172 | 57.14% |
| Observer/observation bias | 246 | 81.73% |
| Publication bias | 215 | 71.43% |
| Reporting/Presentation bias | 166 | 55.15% |
| Researcher/Research bias | 182 | 60.47% |
| Language bias | 109 | 36.21% |
| Measurement bias | 170 | 56.48% |
| Rounding bias | 92 | 30.56% |
| Geographic bias | 173 | 57.48% |
| Cognitive bias | 78 | 25.91% |
| Prejudice bias | 103 | 34.22% |
| Belief bias | 89 | 29.57% |
| Myside bias | 29 | 9.63% |
| Funding bias | 168 | 55.81% |
| Phylogenetic bias | 86 | 28.57% |
| Recall bias | 40 | 13.29% |

**Please express your opinion regarding the severity of the impact of biases on science in general**

| **Severity of impact** | **n** | **Percent** |
| --- | --- | --- |
| High | 103 | 34.22% |
| Medium | 167 | 55.48% |
| Negligible | 9 | 2.99% |
| I do not know | 22 | 7.31% |

**Please express your opinion regarding the severity of the impact of biases on your particular scientific field**

| **Severity of impact** | **n** | **Percent** |
| --- | --- | --- |
| High | 89 | 29.57% |
| Medium | 170 | 56.48% |
| Negligible | 21 | 6.97% |
| I do not know | 21 | 6.98% |

**Please express your opinion regarding the severity of the impact of biases on your own research**

| **Severity of impact** | **n** | **Percent** |
| --- | --- | --- |
| High | 37 | 12.29% |
| Medium | 150 | 49.83% |
| Negligible | 61 | 20.27% |
| I do not know | 43 | 14.29% |
| I do not conduct research | 10 | 3.32% |

**In your opinion, to what extent are the following stages of scientific research prone to biases? (From 1: not prone at all to 5: highly prone)**

| **Stages of research** | **1** | **2** | **3** | **4** | **5** | **Average** | **Median** |
| --- | --- | --- | --- | --- | --- | --- | --- |
| Planning/designing the study | 4.98% | 12.29% | 17.28% | 26.58% | 38.87% | 3.82 | 4 |
| Implementing the study | 3.32% | 21.26% | 32.23% | 25.25% | 17.94% | 3.33 | 3 |
| Analysing the results | 3.99% | 13.62% | 25.91% | 37.21% | 19.27% | 3.54 | 4 |
| Interpreting the results | 1.99% | 8.64% | 15.95% | 34.22% | 39.2% | 4 | 4 |
| Reporting the outcomes | 3.99% | 9.97% | 22.59% | 34.22% | 29.23% | 3.75 | 4 |
| Publishing the outcomes | 4.32% | 10.96% | 20.6% | 27.91% | 36.21% | 3.81 | 4 |

**In your opinion, to what extent are the following research fields affected by biases? (From 1: not affected to 5: highly affected)**

| **Research fields** | **1** | **2** | **3** | **4** | **5** | **Average** | **Median** |
| --- | --- | --- | --- | --- | --- | --- | --- |
| Medical sciences | 1.8% | 10.47% | 20.94% | 32.13% | 34.66% | 3.87 | 4 |
| Cognitive sciences | 1.12% | 9.74% | 32.58% | 27.72% | 28.84% | 3.73 | 4 |
| Behavioural sciences | 0.74% | 5.88% | 19.85% | 39.34% | 34.19% | 4 | 4 |
| Social sciences | 0.36% | 5.44% | 15.22% | 34.78% | 44.2% | 4.17 | 4 |
| Environmental sciences | 2.1% | 10.84% | 36.36% | 32.87% | 17.83% | 3.53 | 4 |
| Ecological sciences | 2.78% | 11.8% | 39.58% | 31.6% | 14.24% | 3.43 | 3 |
| Physiological sciences | 3.35% | 24.16% | 38.29% | 23.79% | 10.41% | 3.14 | 3 |
| Biochemical sciences | 5.91% | 28.41% | 39.48% | 19.19% | 7.01% | 2.93 | 3 |

**In your opinion, to what extent are the following types of scientific publications prone to biases? (From 1: not prone at all to 5: highly prone)**

| **Types of publications** | **1** | **2** | **3** | **4** | **5** | **Average** | **Median** |
| --- | --- | --- | --- | --- | --- | --- | --- |
| Research papers based on observational data | 1.99% | 8.97% | 23.59% | 42.53% | 22.92% | 3.75 | 4 |
| Research papers based on experimental data | 4.65% | 17.94% | 32.89% | 28.57% | 15.95% | 3.33 | 3 |
| Research papers based on modelling | 3.32% | 10.96% | 28.91% | 36.88% | 19.93% | 3.59 | 4 |
| Narrative reviews | 1.99% | 7.97% | 23.92% | 35.55% | 30.57% | 3.85 | 4 |
| Meta-analyses | 4.32% | 19.93% | 36.21% | 25.25% | 14.29% | 3.25 | 3 |

**In your opinion, which methods allow researchers to avoid biases or to minimize their impacts on ecological and environmental research? (Mark all that apply)**

| **Methods** | **n** | **Percent** |
| --- | --- | --- |
| Random choice of experimental units | 221 | 73.42% |
| Haphazard choice of experimental units | 44 | 14.62% |
| Blind collection of data (when observers are not informed about sample origin and/or research hypotheses) | 212 | 70.43% |
| Checking for repeatability of all measurements | 234 | 77.74% |
| Participation in intercalibration exercises | 163 | 54.15% |
| Reporting all results, not only the results that showed statistically significant effects | 267 | 88.7% |
| I do not know | 9 | 2.99% |

**If you conduct research and/or teach/supervise students, please indicate the actions which you have ever performed (mark all that apply).**

| **Actions performed** | **n** | **Percent** |
| --- | --- | --- |
| Thinking about which biases could affect the outcomes of your study | 245 | 81.4% |
| Planning and implementing particular measures to avoid biases in your research | 227 | 75.42% |
| Reporting in your thesis/publications the measures you applied to avoid biases | 184 | 61.13% |
| Discovering biases in scientific manuscripts or publications by others | 147 | 48.84% |
| Teaching students how to avoid biases in scientific research | 170 | 56.48% |
| I do not conduct the research or teach/supervise students | 12 | 3.99% |

**From the definitions provided below (which are all correct), please select those that fit best your personal understanding of bias, as applied to environmental or ecological research. (Select up to three options)**

| **Definitions** | **n** | **Percent** |
| --- | --- | --- |
| Strong inclination of the mind or a preconceived opinion about something | 145 | 48.17% |
| Tendency to search for, interpret, and publish information in a way that confirms one’s pre-existing beliefs or hypotheses | 240 | 79.73% |
| Tendency of a measurement process to over- or under-estimate the value of a parameter | 92 | 30.56% |
| Systematic error in results or inferences that favours one outcome over others | 99 | 32.89% |
| The case when certain organisms, systems, etc. are well studied while others are not | 125 | 41.53% |
| Tendency to perform experiments on organisms or under conditions in which one has a reasonable expectation of detecting statistically signiﬁcant effects | 130 | 43.19% |
| Preferential publication of statistically significant results | 184 | 61.13% |

**Please provide basic information on yourself**

**Age:**

| **Age (years)** | **n** | **Percent** |
| --- | --- | --- |
| Under 20 | 0 | 0% |
| 21–30 | 67 | 21.83% |
| 31–40 | 93 | 30.29% |
| 41–50 | 67 | 21.82% |
| 51–60 | 46 | 14.98% |
| Over 60 | 34 | 11.08% |

**Sex**

| **Sex** | **n** | **Percent** |
| --- | --- | --- |
| Female | 133 | 43.46% |
| Male | 158 | 51.63% |
| Other | 1 | 0.33% |
| Prefer not to say | 14 | 4.58% |

**Counrty where you live now**

| **Country** | **n** | **Percent** |
| --- | --- | --- |
| Argentina | 1 | 0.33% |
| Australia | 3 | 0.97% |
| Brazil | 11 | 3.57% |
| Canada | 13 | 4.22% |
| Chile | 2 | 0.65% |
| China | 8 | 2.6% |
| Colombia | 1 | 0.33% |
| Czech Republic | 4 | 1.3% |
| Denmark | 1 | 0.33% |
| Egypt | 4 | 1.3% |
| Estonia | 2 | 0.65% |
| Finland | 34 | 11.04% |
| France | 7 | 2.27% |
| Germany | 11 | 3.57% |
| Greece | 1 | 0.33% |
| Iceland | 1 | 0.33% |
| India | 1 | 0.32% |
| Indonesia | 2 | 0.65% |
| Iran | 1 | 0.32% |
| Italy | 4 | 1.3% |
| Japan | 2 | 0.65% |
| Lesotho | 1 | 0.32% |
| Mexico | 1 | 0.32% |
| Netherlands | 2 | 0.65% |
| Norway | 6 | 1.95% |
| Pakistan | 2 | 0.65% |
| Poland | 1 | 0.32% |
| Portugal | 2 | 0.65% |
| Russia | 32 | 10.39% |
| Saudi Arabia | 2 | 0.65% |
| Slovakia | 2 | 0.65% |
| Slovenia | 2 | 0.65% |
| South Africa | 2 | 0.65% |
| Spain | 7 | 2.27% |
| Sweden | 9 | 2.92% |
| Switzerland | 4 | 1.3% |
| United Kingdom | 4 | 1.3% |
| United States | 112 | 36.36% |
| Uzbekistan | 1 | 0.32% |
| Zimbabwe | 2 | 0.65% |

**Your primary affiliation**

| **Primary affiliation** | **n** | **Percent** |
| --- | --- | --- |
| College, university or other educational institution | 211 | 68.51% |
| Research institute or similar organisation | 73 | 23.7% |
| Commercial company | 7 | 2.27% |
| Other | 17 | 5.52% |

**Stage of a career**

| **Stage of a career** | **n** | **Percent** |
| --- | --- | --- |
| College or university student | 31 | 10.3% |
| PhD student | 54 | 17.94% |
| Postdoctoral researcher | 42 | 13.95% |
| Research fellow, lecturer, professor, or similar | 174 | 57.81% |

**Duration of professional activity after graduation**

| **Duration of scientific activity** | **n** | **Percent** |
| --- | --- | --- |
| Have not graduated yet | 37 | 12.01% |
| <5 years | 71 | 23.05% |
| 5–10 years | 53 | 17.21% |
| 10–20 years | 50 | 16.24% |
| >20 years | 97 | 31.49% |

**Are you currently involved in teaching? (Mark all that apply)**

| **Involvement in teaching** | **n** | **Percent** |
| --- | --- | --- |
| This question is not applicable to my stage of scientific career | 27 | 8.77% |
| I teach at a university (or similar) on the regular basis | 100 | 32.47% |
| I teach at a university (or similar) from time to time | 73 | 23.7% |
| I supervise Master’s and/or PhD students and/or postdoctoral researchers | 118 | 38.31% |
| I do not teach | 92 | 29.87% |

**How many papers did you publish in peer-reviewed international scientific journals during the past three years?**

| **Papers published** | **n** | **Percent** |
| --- | --- | --- |
| 0 | 54 | 17.53% |
| 1–5 | 128 | 41.56% |
| 6–15 | 80 | 25.97% |
| 16–25 | 28 | 9.09% |
| >25 | 18 | 5.85% |

**How many manuscripts did you review for international journals during the past three years?**

| **Manuscripts reviewed** | **n** | **Percent** |
| --- | --- | --- |
| 0 | 74 | 24.03% |
| 1–5 | 97 | 31.49% |
| 6–15 | 83 | 26.95% |
| 16–25 | 24 | 7.79% |
| >25 | 30 | 9.74% |

**Do you think that this questionnaire is likely to produce a biased outcome?**

| **Does this questionnaire produce a biased outcome?** | **n** | **Percent** |
| --- | --- | --- |
| Yes | 140 | 45.75% |
| No | 18 | 5.88% |
| I do not know | 148 | 48.37% |
